# Analyzing the seasonality of tuberculosis case notifications in the UK, 2000–2018

**DOI:** 10.1017/S095026882400092X

**Published:** 2024-10-01

**Authors:** Lisa Glaser, Ross Harris, Tehreem Mohiyuddin, Jennifer A. Davidson, Sharon Cox, Colin N. J. Campbell

**Affiliations:** 1 Travel Health, Zoonosis, Emerging Infections of Pandemic Potential and Respiratory & Tuberculosis Division, UK Health Security Agency, London, UK and; 2 Statistics Production Division, UK Health Security Agency, London, UK

## Abstract

Globally, there is seasonal variation in tuberculosis (TB) incidence, yet the biological and behavioural or social factors driving TB seasonality differ across countries. Understanding season-specific risk factors that may be specific to the UK could help shape future decision-making for TB control. We conducted a time-series analysis using data from 152,424 UK TB notifications between 2000 and 2018. Notifications were aggregated by year, month, and socio-demographic covariates, and negative binomial regression models fitted to the aggregate data. For each covariate, we calculated the size of the seasonal effect as the incidence risk ratio (IRR) for the peak versus the trough months within the year and the timing of the peak, whilst accounting for the overall trend. There was strong evidence for seasonality (*p* < 0.0001) with an IRR of 1.27 (95% CI 1.23–1.30). The peak was estimated to occur at the beginning of May. Significant differences in seasonal amplitude were identified across age groups, ethnicity, site of disease, latitude and, for those born abroad, time since entry to the UK. The smaller amplitude in older adults, and greater amplitude among South Asians and people who recently entered the UK may indicate the role of latent TB reactivation and vitamin D deficiency in driving seasonality.

## Introduction

Tuberculosis (TB) is a respiratory infection that typically affects the lungs (pulmonary TB) but can also affect other organs (extra-pulmonary TB). A relatively small proportion of people who become infected (5–10%) will develop active TB disease during their lifetime. Disease may develop as a result of recent infection indicating ongoing TB transmission, or due to the reactivation of a remotely acquired latent TB infection (LTBI).

Seasonal variation in TB incidence has been reported in many countries [1–9]. In the UK, seasonality has been identified at national and local levels, with TB notifications peaking in the spring and summer and a trough in the winter [10]. Interestingly, TB seasonality appears to be significantly delayed in comparison to acute respiratory disorders, for which notifications peak around January or February [8].

Existing hypotheses for TB seasonality include increased winter transmission due to indoor activities leading to prolonged periods of close contact [1, 9], and an increased infection risk due to lowered immune response associated with decreased vitamin D levels [10–13]. The period between infection, onset of symptoms, and the delay to diagnosis, is hypothesized to cause a peak in TB notifications in the spring and summer.

Although previous studies identified TB seasonality in the UK, the factors associated with this remain poorly understood. The UK has a low incidence of TB, with active TB largely driven by migration of individuals from countries with a high burden of disease, accounting for approximately three-quarters of all notifications [14, 15]. Understanding season-specific risk factors that may be specific to the UK could help shape future decision-making for the control of TB, optimize seasonal preparedness and raise awareness to improve swift detection. This study aims to quantify the extent of seasonal variation in TB notifications in the UK between 2000 and 2018 and explore the possible factors contributing to this seasonality.

## Methods

### Data sources

Data for active TB notifications between 1 January 2000 and 31 December 2019 in England, Wales, and Northern Ireland was obtained from the Enhanced Tuberculosis Surveillance system, and in Scotland from the Enhanced Surveillance of Mycobacterial Infections system. Notifications include people who are resident or treated in the UK.

Notification was based on microbiological confirmation for *Mycobacterium tuberculosis* complex through a positive culture, PCR, microscopical examination or histology result. In the absence of microbiological confirmation, the notification was based on the clinician’s judgement that the patient’s clinical signs, radiological signs and/or symptoms were compatible with TB, and the decision was made to treat the patient with a full course of anti-TB therapy.

### Dataset preparation

To assess factors that may be associated with different seasonality or trends, we included the following socio-demographic covariates for analysis: sex, age group, ethnicity, place of birth (UK or abroad), latitude of residence in the UK, UK region, rural-urban classification, site of disease, first line drug resistance profile, Index of Multiple Deprivation (IMD) decile, social risk factors (SRFs; defined as: homelessness, imprisonment, alcohol misuse and drug misuse), previous TB diagnosis, and time since entry into the UK (for people born abroad only). The latter was combined with place of birth to simplify analysis.

Latitude data was categorized into two strata according to the effects of latitude on solar ultraviolet radiation across the UK. Monitoring of solar ultraviolet radiation and illuminance levels at six different latitude sites in Great Britain indicated a lower mean noontime irradiance further north, however irradiance levels showed reduced variance for latitudes above 52° N [16]. Latitude data was therefore grouped as 50.0–51.9° N and 52.0+° N. Latitude data for Scottish cases was only available until 2017, and could not be validated for an additional 44 cases; overall, 721 cases were excluded from this analysis.

TB notifications were assigned an IMD 2019 rank based on Lower Super Output Area (LSOA) of residence using the 2011 census. The LSOAs were assigned deprivation categories by sorting them from most to least deprived using the IMD 2019 rank, and then divided into deciles [17].

Two date definitions were used to classify the earliest evidence of TB to better understand the biological, social and environmental factors associated with seasonality. Firstly, we defined the earliest disease date as the earliest date of symptom onset, specimen collection, diagnosis, presentation to health services, or start of treatment. We then defined the earliest clinical date as the earliest date of specimen collection, diagnosis, presentation to health services or start of treatment. There was a high completeness (>90%) for symptom onset, diagnosis and treatment start dates, and a lower completeness (>60%) for specimen collection and presentation to health services dates. Overall, the date hierarchy used to create the earliest disease and clinical dates was well maintained; symptom onset was most commonly used as the earliest disease date, followed by specimen date if the former was unknown, and specimen date was most commonly used as the earliest clinical date, followed by date of diagnosis.

TB cases may be notified late onto the surveillance system. To ensure no cases occurring within a year were missed, only years for which data for the full year was available were included. As such, only cases with an earliest disease or clinical date, as defined above, occurring between 2000 and 2018 were included for analysis, as some cases for later years may not have yet been reported at the time of analysis.

### Statistical analysis

A time-series analysis was performed on both the earliest disease date as well as the earliest clinical date. For each date, cases were aggregated by year and month, and according to the above-mentioned socio-demographic covariates, to investigate overall and covariate-specific annual trends and monthly patterns. Monthly data was used to investigate the seasonality of TB, where the seasons in the UK are defined as spring (March, April, May), summer (June, July, August), autumn (September, October, November) and winter (December, January, February).

Negative binomial regression models were fitted to the resulting monthly case counts. The models included linear and quadratic temporal trend components, and sine and cosine terms with a period of twelve months to incorporate annual seasonal trends [18], whilst also accounting for overdispersion in the data. The monthly counts were also weighted to the length of the month by including an offset in the log-linear model to scale all monthly counts to a rate per 30 days [19]. For each socio-demographic covariate, the size of the seasonal effect (amplitude) was calculated as the incidence risk ratio (IRR) for the peak versus the trough month within the year, as well as the timing of the peak (shift), whilst accounting for the overall trend.

Differences in seasonal amplitude (measured by the IRR) across subgroups were assessed within a full multivariable model for covariates which demonstrated significant differences during univariate analysis. The multivariable model adjusted for all covariates included in the model and allowed for differences in the overall trend and seasonal patterns. The importance of overall seasonal effects and differences in seasonal effects across subgroups were then assessed by removing the relevant covariate from the model and comparing with the full model via likelihood ratio tests.

Extra-pulmonary TB is often the result of more advanced disease, and its presentation is therefore strongly influenced by the host’s immunologic capacity, which may make it more sensitive to seasonal immunosuppression in winter. In the UK, children are less likely to develop extra-pulmonary TB [20], yet young age is a known risk factor for increased TB seasonality [1]. The interaction effects between age group and site of disease were therefore further assessed in the same way. The interaction effects for ethnic group and latitude were also assessed to investigate the potential impacts of increased vitamin D deficiency in higher latitudes, acknowledging that some ethnicities have a higher risk of vitamin D deficiency. Results were considered significant when *p* ≤ 0.05. Data management and analyses were performed using Stata 17 (College Station, TX: StataCorp LLC).

## Results

**Figure 1.**
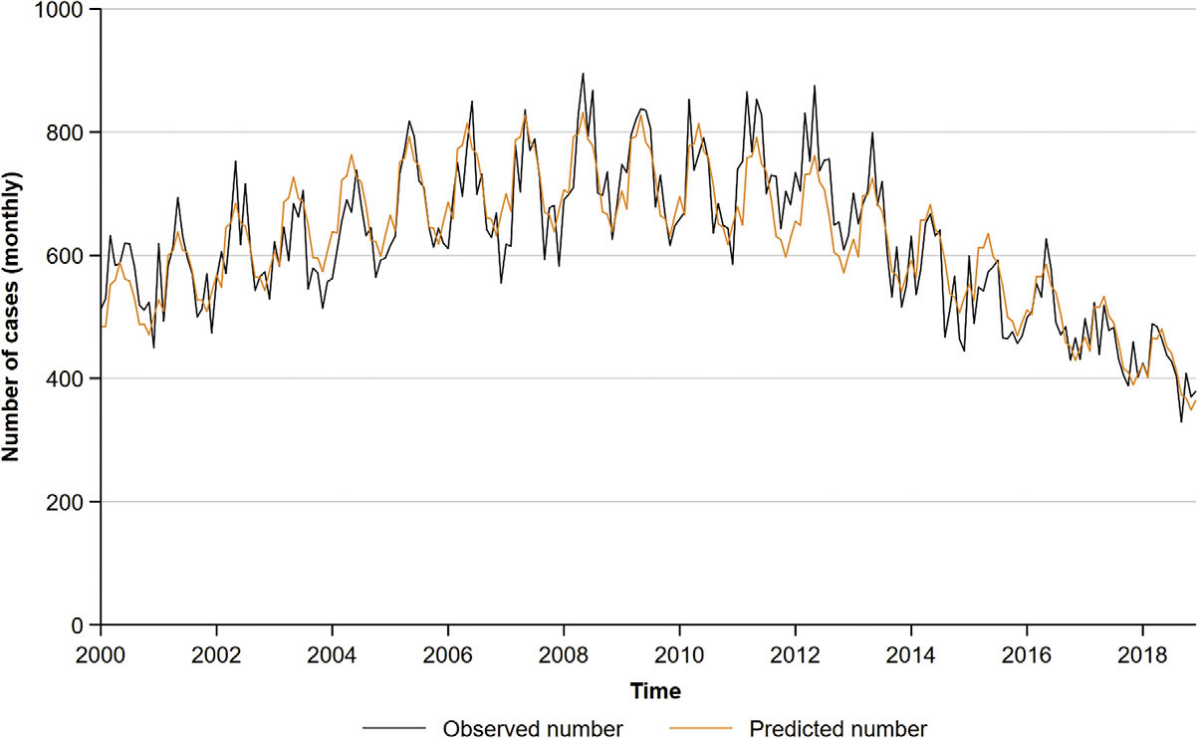
Negative binomial regression model fitted onto the monthly aggregate tuberculosis notifications by earliest clinical date.

A total of 152,424 active notifications in the UK were considered for analysis. Overall, the number of cases increased between 2000 (*n* = 6,686) and 2011 (*n* = 8,919) and decreased thereafter (2018: 5,036) (Figure 1). A higher proportion of cases were notified over the spring and summer months (May-July: 28%) compared to the winter months (December-February: 23%). The estimated IRR for the peak versus trough months during this time period was 1.27 (95% CI 1.23–1.30; p<0.001), showing strong evidence for seasonality. The annual peak was estimated to occur at the beginning of May.

**Figure 2.**
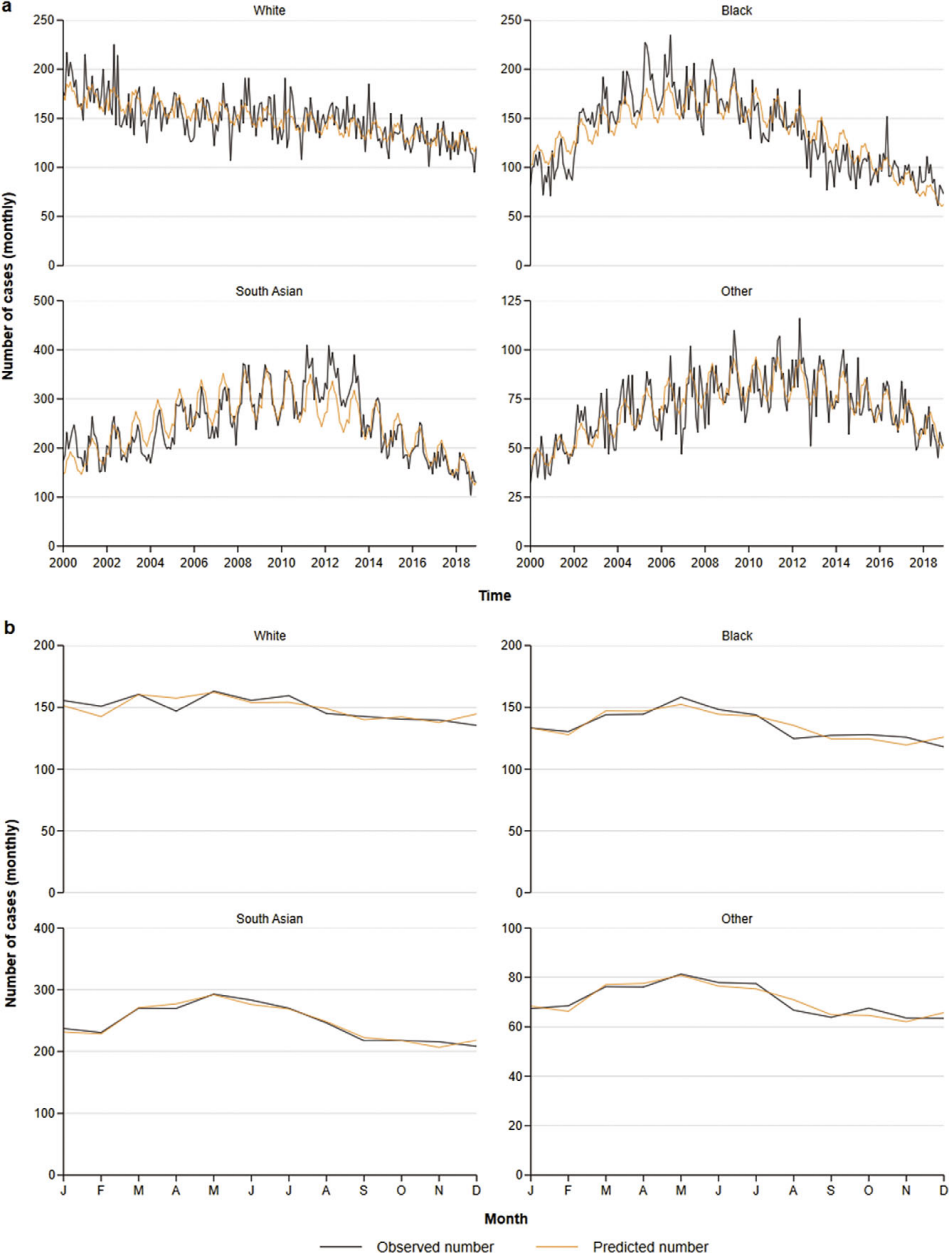
Negative binomial regression models fitted onto the total monthly aggregate tuberculosis notifications (a) and onto the mean monthly tuberculosis case counts (b) by earliest clinical date and ethnicity. Note axes differ across graphs to better visualize the data being shown.

**Figure 3.**
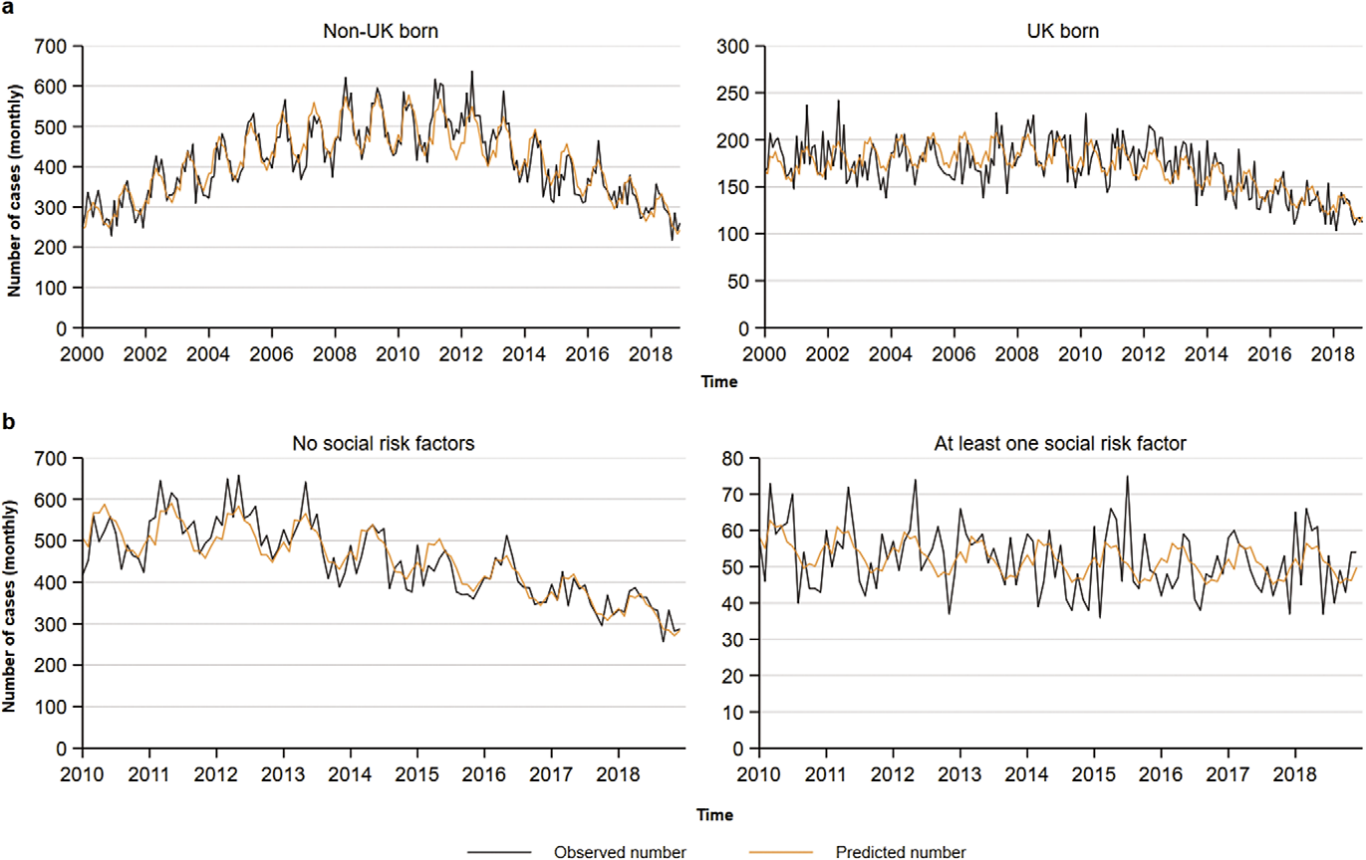
Negative binomial regression models fitted onto the monthly aggregate tuberculosis notifications by earliest clinical date and by place of birth (a) or presence of social risk factors (b). Data for social risk factors was only available for cases from 2010. Note axes differ across graphs to better visualize the data being shown.

Notifications among people of white ethnicity decreased steadily throughout the study period from 2,191 in 2000 to 1,441 in 2018. In contrast, notifications among people of black and south Asian ethnicities increased from 2000 to 2006 and 2012, respectively, and decreased thereafter (Figure 2). Similarly, TB notifications among people born abroad increased between 2000 and 2011, and decreased thereafter, whereas the trend remained relatively stable among people born in the UK until 2011, with a smaller decline thereafter (Figure 3). When comparing people with SRFs with people without a SRF; case notifications decreased from 2012 to 2018 in the latter, however these remained relatively stable among people with a SRF in the same time period (Figure 3).

The model fitting identified overdispersion in the data, likely due to variations in the seasonal pattern or magnitude overtime, resulting in a poorer overall model fit. When fitted to the mean monthly case counts, however, the models appeared to fit the average seasonal pattern quite well (Figure 2). Analysis of seasonality for both the earliest disease and earliest clinical dates is presented in Supplementary Table S1.

**Figure 4.**
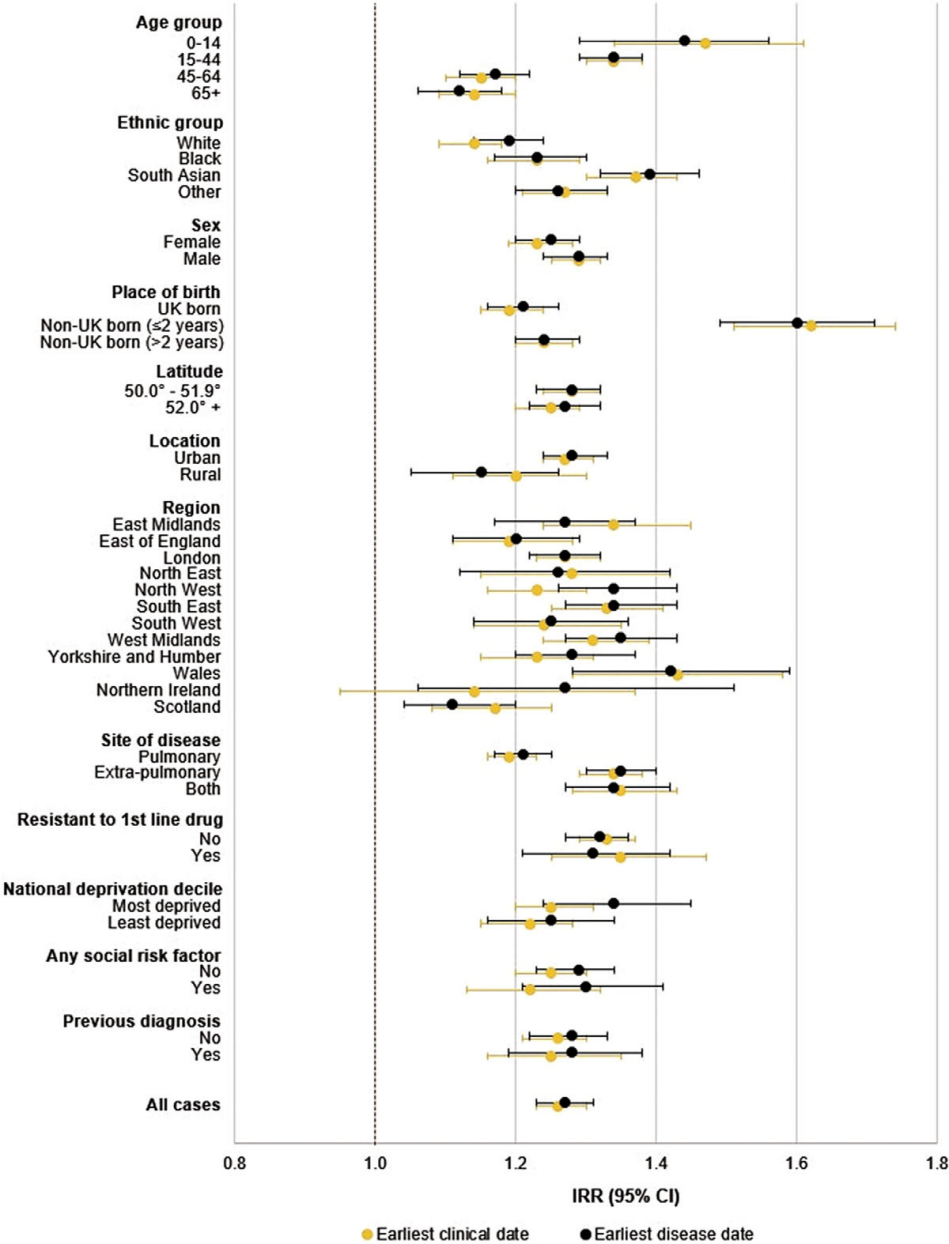
Size of the tuberculosis seasonal effect (amplitude), calculated as the incidence risk ratio (IRR), and 95% confidence interval error bars, for the earliest clinical date and earliest disease date by covariate.

Overall, the size of the seasonal effect was significant at IRR 1.26 (95% CI 1.23–1.30) by earliest clinical date. The size of the seasonal amplitude differed significantly within subgroups: those aged <15 years (IRR 1.47 [1.34–1.61]) had a greater seasonal amplitude compared to the older ages (45–64 years: 1.15 [1.10–1.20]; 65+ years: 1.14 [1.09–1.20]). Seasonal amplitude was also greater among people of south Asian ethnicity (1.37 [1.30–1.43]) compared to other ethnicities, people living in lower latitudes (50.0–51.9° N: 1.28 [1.24–1.32]; 52.0° N+: 1.25 [1.20–1.29]), people with extra-pulmonary disease (extra-pulmonary: 1.34 [1.29–1.34]; pulmonary: 1.19 [1.16–1.23]) and people who entered the UK within the two years prior to their TB diagnosis (≤2 years: 1.62 [1.51–1.74]; >2 years: 1.24 [1.20–1.28]). There were no significant differences in seasonal amplitude between earliest disease and earliest clinical date (Supplementary Table S1; Figure 4).

**Figure 5.**
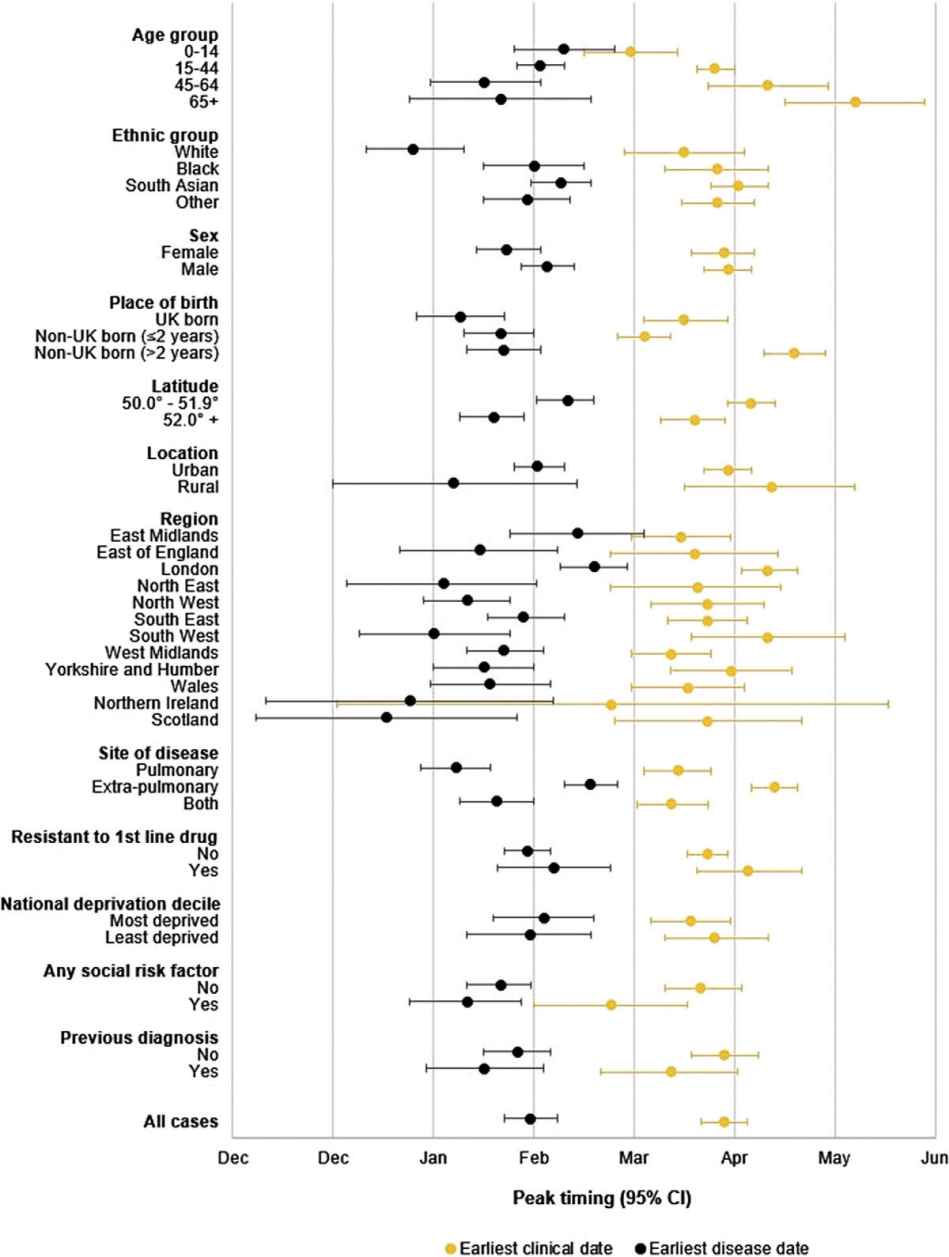
Timing of the tuberculosis seasonal peak (shift) and 95% confidence interval error bars, for the earliest clinical date and earliest disease date by covariate.

Overall, the timing of the seasonal peak was estimated to occur on 26 April [95% CI 19 April–3 May] by earliest clinical date. The timing differed significantly within some subgroups: the seasonal peak occurred later in the year for those aged >65 years (4 June [14 May–25 June]) compared to those aged <15 years (29 March [15 March–12 April]). The peak also occurred later in the year among people who entered the UK more than two years prior to their TB diagnosis (>2 years: 17 May [8 May–26 May)]; ≤2 years: 2 April [25 March–10 April]), people living in lower (50.0–51.9° N: 4 May [27 April–11 May]; 52.0° N+: 17 April [7 April–26 April]), and people with extra-pulmonary disease (extra-pulmonary: 11 May [4 May–18 May]; pulmonary: 12 April [2 April–22 April]) (Supplementary Table S1; Figure 5).

The seasonal peak differed between the earliest disease date (28 February [20 February–7 March]) and earliest clinical date (26 April [19 April–3 May]). These differences were also apparent within some covariates; for instance, there were no significant differences in the timing of the peak across age groups by earliest disease date. The seasonal peak by earliest disease date was estimated to occur earlier in the year among people of white ethnicity (24 January [10 January–8 February]) compared to other ethnicities (Supplementary Table S1; Figure 5).

Covariates which demonstrated significant differences in seasonal amplitude during univariate analysis included age group, ethnic group, place of birth, latitude, site of disease and UK region. These were further analyzed within a full multivariable model except for UK region, which was excluded as small sample sizes, resulting from the inclusion of a categorical variable with many levels, resulted in confidence intervals being too wide for reliability of results. The multivariable model showed no significant differences in estimates compared to the univariable model (Supplementary Table S1).

Interactions between ethnicity and latitude, as well as between age group and site of disease, were further analyzed. Whilst the seasonal amplitude by ethnicity didn’t significantly vary by latitude, the timing of the peak appeared to occur earlier in the year in higher latitudes across all ethnicities. The seasonal amplitude was also highest among those aged <15 years with both pulmonary and extra-pulmonary disease (1.58 [1.25–2.00]), and lowest among those aged >44 years with pulmonary disease (1.19 [1.14–1.24]). The timing of the peak by earliest disease date occurred earlier in the year with increasing age, whereas the peak occurred later in the year with increasing age by earliest clinical date (Supplementary Table S2).

## Discussion

Our study found a clear, consistent annual seasonality in TB notifications in the UK, which were highest in the early summer and lowest in the late winter. Analysis of the seasonal amplitude across covariates identified a significantly greater seasonal effect in young individuals, people of South Asian ethnicity, people born abroad (especially those who recently entered the UK) and people with extra-pulmonary disease. The timing of the seasonal peak occurred earlier in the year in people aged <15 years, of white ethnicity, born in the UK, living in higher latitudes or with pulmonary disease. The delay between symptom onset and diagnosis was greater for older individuals, people of white ethnicity and people born abroad who entered the UK >2 years prior to their diagnosis.

Children aged <15 years had a significantly greater seasonal amplitude compared to older individuals, which aligns with studies undertaken in the USA [1]. Young children are more likely to acquire active TB through recent transmission, which may be heavily influenced by season. Conversely, active TB in older individuals likely results from LTBI reactivation, which is increasingly influenced by the host’s immune system and could explain the lower seasonality in these groups.

TB seasonality may also be indicative of an association with meteorological factors; previous studies [21–25] found that high levels of ultraviolet light may hamper bacterial growth and promote synthesis of vitamin D [26]. The potential protective effects of vitamin D against developing active TB disease have been widely investigated, and its deficiency has been epidemiologically associated with TB [11, 27–29] and shown to suppress the immune response specific to TB disease [30]. As cutaneous synthesis is the main source of vitamin D, the duration of sun exposure may influence TB incidence. Low vitamin D levels in the winter among individuals with a reduced exposure to sunlight could result in increased incidence through reactivation of LTBI and spring surges in notifications. In high latitude regions such as the UK, lowered sun exposure has been associated with a higher prevalence of vitamin D deficiency particularly among people with darker skin who have a natural barrier to the already low ultraviolet radiation penetrating the skin [13]. This effect has been hypothesized to be enhanced among recent immigrants of TB endemic countries moving into more northern latitudes, who may be at increased risk for LTBI reactivation. This has previously been referred to as an ‘acquired immune deficiency of immigration’ [10]. People of South Asian ethnicity appeared to have the highest seasonal amplitude in the UK. Behavioural aspects may further influence this seasonal pattern in the UK [12].

The effects of latitude on TB seasonality have been described in countries including Pakistan [2], India [3], China [4], and Australia [5], but did not appear to be significant in the USA [1] or Japan [6]. Our study showed latitude had a minimal yet significant effect on TB seasonality in the UK, with a greater seasonal amplitude observed in lower latitudes. It is possible that the high ethnic diversity in London results in a higher proportion of vitamin D deficiency in its population [31, 32] driving a higher seasonality in lower latitudes. Higher humidity may also influence the smaller seasonality seen in higher latitudes, as this has been associated with a lower transmission of aerosols [33].

A study in Japan suggested that a peak in spring could also be due to a delay in diagnosis in wintertime disease [34]. During the winter, early TB disease may be misdiagnosed due to non-specific symptoms resembling a viral upper respiratory infection. Longer delays may lead to cases of advanced disease, or extra-pulmonary TB, peaking even later. A longer diagnostic delay could also lead to a smaller seasonal peak in incidence due to the greater variation in time between infection and onset of disease. This effect may be pronounced across age groups; microbiological confirmation tends to be low among children compared to adults, as children are often diagnosed via contact tracing or based on clinical suspicion [20]. This results in a shorter diagnostic delay which may influence the greater seasonal amplitude observed among children. Concerned parents may also be more likely to seek earlier healthcare for young children than they are for themselves. On the other hand, older individuals may be more often misdiagnosed due to multi-morbidity, where diagnostic delays may result in a weakened seasonal peak.

Whilst the earliest disease date is primarily influenced by pathogen microbiology and host immunity, the earliest clinical date is affected by a range of external factors including environmental, human behaviour and healthcare delays. Differences in the timing of the peak were observed among people born abroad, who may infer a greater suspicion of TB among healthcare workers, yet the longer these individuals reside in the UK, the risk of developing TB disease through recent transmission decreases, potentially leading to lower suspicion and diagnostic delays. In contrast, with Europe’s TB burden being among the lowest globally as well as having the fastest decline compared to other WHO Regions [35], there is a lower suspicion of TB among people born in the UK or of white ethnicity, who are generally regarded as having a lower risk of TB disease. Furthermore, the higher proportion of older individuals in this group may further contribute to a delay in TB notification.

This study benefits from the availability of data collected over a prolonged time period. Long term trends, however, may partly simply reflect changes in the underlying population over time; the UK population saw a steady increase between 2000 and 2019. The South experienced the greatest change, especially London, with a 23% increase in the population during this time period, compared to a slower increase in the North (the North East only increased by 4%) [36]. This may have been partly driven by a growing foreign-born population, which reached a proportion of 14% in 2019, up from just under 9% in 2004 [37]. Furthermore, whilst there are no official time series population estimates by ethnicity, experimental statistics indicate that the ‘White British’ population in Great Britain shrank from 89% in 2001 to 79% in 2019, whilst all other ethnicities more than doubled in the same time period [32]. These population trends align with the increasing TB notifications in lower latitudes and across ethnicities until 2012, but decreasing notifications thereafter are indicative of alternative factors affecting TB trends, and coincides for instance with the introduction of the UK pre-entry TB screening for migrants from countries of high endemicity [38].

The older adult population in the UK, on the other hand, has been slowly increasing, especially those aged ≥65 years, which increased by 38% between 2000 and 2019, whilst the younger population has remained fairly stable overtime [36]. Conversely, TB notifications declined in those aged ≥65 years, but increased among those aged 15–44 years until 2012, and decreased thereafter, most likely reflecting the higher proportion of cases born abroad, rather than the underlying UK population. Whilst analysis of the TB incidence rate could have provided further insight into the effects of population trends, population data is inconsistent and was not available for all sub-groups. A further limitation is the timing of the trough within the year being estimated by using sine and cosine terms with a period of twelve months based on the calculation of the peak, as this may obscure the exact shape of seasonal patterns. However, there was no evidence of non-standard seasonal patterns in the data when an individual effect for each month was added.

The findings of this study should be considered in the development of future TB control strategies in the UK to address and minimize the seasonal increase of TB cases. Clinical teams should be aware of high-risk groups, and have an understanding of how seasonal trends may differ across groups to improve early detection. The provision of individualized advice to people at higher risk of vitamin D deficiency, people born in countries with a high burden of TB, and to those with diagnosed LTBI, should also be considered. This may include vitamin supplementation or antibiotic therapy as appropriate.

## Conclusions

TB notifications display seasonality in the UK, being highest in the early summer and lowest in the late winter. A smaller seasonal amplitude in older adults, and a greater amplitude in people of South Asian ethnicity and people born outside the UK who recently entered the UK may be suggestive of the higher influence of seasonality on recent transmission than on disease resulting from reactivation of latent infection, as well as of the impact of vitamin D deficiency. This study highlights the differences in factors affecting TB seasonality across countries and differentiates between the biological and the behavioural or social factors which impact TB seasonality in the UK, and how these may change in the light of continued national and global TB control.

## Supporting information

Glaser et al. supplementary material Glaser et al. supplementary material

## Data Availability

The data underlying this article are available in the article and in its online supplementary material.

## Supplementary material

For supplementary material accompanying this paper visit http://dx.doi.org/10.1017/S095026882400092X. click here to view supplementary material
